# Supplementary material for: Metabolic control in patients with type 2 diabetes mellitus in a public hospital in Peru: a cross-sectional study in a low-middle income country
Source: PeerJ. 2016 Oct 13;4:e2577. doi: 10.7717/peerj.2577 (PMC5068371; doi:10.7717/peerj.2577)
Supplement: Table S1 [file peerj-04-2577-s001.docx]

| Categorical Score | Description |
| --- | --- |
| 1. Inactive | - No activity is reported or - Some activity is reported but not enough to meet Categories 2 or 3 |
| 1. Minimally active | Any of the following three criteria:   - 3 or more days of vigorous activity of at least 20 minutes per day OR - 5 or more days of moderate-intensity activity or walking at least 30 minutes per day OR - 5 or more days of any combination of walking, moderate-intensity or vigorous intensity activities achieving a minimum of at least 600 MET-minutes/week |
| 1. HEPA(health enhancing physical activity) | Any of the following two criteria:   - Vigorous intensity activity on at least 3 days and accumulating at least 1500 MET-min/week OR - 7 or more days of any combination of walking , moderate –intensity, vigorous activities achieving a minimum of at least 3000 MET-minutes/week |

*Supplementary Table S1*

HEPA: Health enhancing physical activity

MET:Metabolic equivalent
